# Invasive slug populations (*Arion vulgaris*) as potential vectors for *Clostridium botulinum*

**DOI:** 10.1186/s13028-014-0065-z

**Published:** 2014-10-03

**Authors:** Kristine Gismervik, Torkjel Bruheim, Liv M Rørvik, Solveig Haukeland, Ida Skaar

**Affiliations:** Norwegian Veterinary Institute, Pb 5695 Sluppen 7485 Trondheim/Pb 750 Sentrum, Oslo, NO-0106 Norway; Norwegian University of Life Sciences, School of Veterinary Science, Pb 8146 dep, Oslo, NO-0033 Norway; Norwegian Institute for Agricultural and Environmental Research, Fr. A. Dahlsvei 20, Ås, NO-1430 Norway

**Keywords:** Slug invasion, Clostridium botulinum, Botulism, Arion vulgaris, Bacterial vector, Silage contamination, qPCR

## Abstract

**Background:**

Norwegian meadows, including those for silage production, are recently found heavily invaded by the slug *Arion vulgaris* in exposed areas. As a consequence, large numbers of slugs might contaminate grass silage and cause a possible threat to animal feed quality and safety. It is well known that silage contaminated by mammalian or avian carcasses can lead to severe outbreaks of botulism among livestock. Invertebrates, especially fly-larvae (Diptera), are considered important in the transfer of *Clostridium botulinum* type C and its toxins among birds in wetlands. *C. botulinum* form highly resistant spores that could easily be consumed by the slugs during feeding. This study aimed to determine whether *Arion vulgaris* could hold viable *C. botulinum* and enrich them, which is essential knowledge for assessing the risk of botulism from slug-contaminated silage. Slug carcasses, slug feces and live slugs were tested by a quantitative real-time PCR (qPCR) method after being fed ≅ 5.8 × 10^4^ CFU *C. botulinum* type C spores/slug.

**Results:**

Low amounts of *C. botulinum* were detected by qPCR in six of 21 slug carcasses with an even spread throughout the 17 day long experiment. Declining amounts of *C. botulinum* were excreted in slug feces up to day four after the inoculated feed was given. *C. botulinum* was only quantified the first two days in the sampling of live slugs. The viability of *C. botulinum* was confirmed for all three sample types (slug carcasses, slug feces and live slugs) by visible growth in enrichment media combined with obtaining a higher quantification cycle (Cq) value than from the non-enriched samples.

**Conclusions:**

Neither dead nor live invasive *Arion vulgaris* slugs were shown to enrich *Clostridium botulinum* containing the neurotoxin type C gene in this study. Slugs excreted viable *C. botulinum* in their feces up to day four, but in rapidly decreasing numbers. *Arion vulgaris* appear not to support enrichment of *C. botulinum* type C.

## Background

Populations of the invasive slug *Arion vulgaris* (erroneously often referred to as *A. lusitanicus*) are spreading rapidly and invade gardens, vegetable crops and meadows including those for silage production [1–3]. Dense populations of more than 50 slugs per square meter have been recorded in wildflower strips and meadows [4]. As a consequence, large numbers of slugs might contaminate grass silage and cause a possible threat to animal feed quality and safety.

It is well known that silage contaminated by mammalian or avian carcasses can lead to severe outbreaks of botulism in livestock [5–7]. One contaminated carcass can be sufficient to cause the death of hundreds of cattle fed mixed rations [8] due to the highly potent neurotoxins (BoNT A-F) produced by *Clostridium botulinum* [9,10]. The neurotoxins block the acetylcholine release at the motor nerve-endings resulting in flaccid paralysis. Death is often caused by respiratory paralysis and circulatory failure. Despite the shared ability to produce neurotoxins, *C. botulinum* is a very heterogeneous species divided phylogenetically and physiologically into four different groups (I-IV). Group IV consists of *Clostridium argentinense* (previously named *C. botulinum*), known for BoNT G production [10]. Type C and D toxin producing strains of *C. botulinum*, belonging to group III, are considered the most common cause of animal botulism [5,11]. Of the other toxin producing strains (A, B, E and F) associated with human botulism [12], type A and B have been reported in bovine botulism outbreaks [5,11].

*C. botulinum* produces highly resistant spores that are widely distributed in soil, dust, animal feces and aquatic environments [9,13,14]. Anaerobic conditions and a suitable growth medium are required for germination and toxin production. Some invertebrate carcasses can satisfy these requirements for *C. botulinum* growth [9,15]. In addition, invertebrates are not susceptible to the toxin and may even concentrate toxins when feeding on decaying carcasses. Fly-larvae (Diptera) feeding on decaying carcasses are considered important in the transfer of *C. botulinum* type C and its toxins among birds in wetlands [15–17]. These highly toxic maggots perform cascades of intoxications when birds consuming maggots receive botulism and die. The carcasses attract flies for egg laying, resulting in an increase of toxic maggots that could lead to avian botulism epizootics [18]. Toxin laden maggots have been reported as a suspected source of botulism in ruminants, although other sources like vertebrate carcasses in drinking water could not be excluded [19,20].

Invasive *A. vulgaris* slugs live in close contact with soil and have a wide diet feeding on various fresh and decaying plants but also animal excrements and waste including carcasses from both invertebrates and vertebrates [1]. Although prevalence studies are missing, the feeding habits of slugs make them quite likely to come in contact with and consume *C. botulinum*. This study aimed to determine whether slugs could hold viable *C. botulinum* and enrich them, essential knowledge for assessing the risk of botulism from slug-contaminated silage. After feeding slugs *C. botulinum* spores, slug carcasses, slug feces and live slugs were tested for *C. botulinum*.

## Materials and methods

### Slugs

In order to secure no presence of *C. botulinum* in the starting population, laboratory hatched *A. vulgaris* were used in the experiment. Eggs were collected in September 2012 from South-East Norway (Røresand), from a location with undisturbed vegetation and with no use of molluscicides. The eggs hatched in September and October at 16 ± 2°C. Slugs were kept in plastic containers on moistened tissue paper and fed sliced carrot, Chinese cabbage and a commercial piglet feed for protein enrichment (Format Kvikk 160, Felleskjøpet Agri, Norway). The feeding experiment started in April 2013. To ensure a fast ingestion of the inoculated feed, the slugs were starved 65 h prior to the onset of the feeding experiment. Slugs used in the experiment were on average 4.5 g (range 3.4-5.8 g). The pH of five scalpel chopped slugs, starved for 48 h, was measured later on using pH-indicator strips (Merck KGaA, Darmstadt, Germany).

### Spore production and quantification

A spore suspension of *C. botulinum* type C was produced by inoculating strain CCUG 7970 (Culture Collection University of Göteborg, Sweden) into eight tubes of 9 ml freshly made tryptose-peptone-glucose-yeast extract (TPGY) broth (5% Tryptone, Becton, Dickinson and company (BD), Sparks, USA, 0.5% Proteose Peptone, BD, 0.4% glucose, Merck, 2% yeast extract, BD, 0.1% starch, Merck, 0.1% l-cystein-HCl and 0.14% NaHCO_3_) [21]. The tubes were heated for 5 min at 70°C and incubated for one week at 37°C under anaerobic conditions (GENbag, bioMerieux, Marcy l’Etoile, France). The cultures were centrifuged and washed as described by Lindberg *et. al.* [22] to remove debris from the spore suspension. The spore suspension was stored at 4 ± 2°C until use, and the viability and purity was determined by plating onto blood agar followed by both aerobic and anaerobic incubation at 37 ± 1°C for 48 h.

The enumeration of the spore suspension was conducted by plating two serial dilutions onto blood agar followed by incubation at 37°C under anaerobic conditions for 72 h. The serial dilutions was frozen immediately at −20°C in portions of one ml in 1.5 ml tubes (Eppendorf AG, Hamburg, Germany) in order to create standard curves in quantitative real-time PCR (qPCR) as later described. The qPCR was used to quantify *C. botulinum* spores given each slug in the experiment. To test the accuracy, another serial dilution of the spore suspension followed by plating on blood agar as previously described was performed the first day of the experiment.

### Preparation of inoculated feed

One cm thick discs of cucumber (*Cucumis sativus*) were sliced and kept in a refrigerator for 48 h to gain a more dry texture capable of some liquid absorption. Small quadratic pieces were prepared from the outer cucumber layer, with the bottom of each piece consisting of peel to prevent penetration of fluid. A small well was sliced by scalpel into each cucumber piece. To each 0.5 g piece of cucumber 0.04 ml of the *C. botulinum* spore suspension was added. Slugs given one piece of inoculated feed received ≅ 5.8 (SD ± 0.1) × 10^4^ CFU *C. botulinum*/ slug determined by qPCR, which was in agreement with the cultivation.

### The feeding experiment

**Figure 1:**
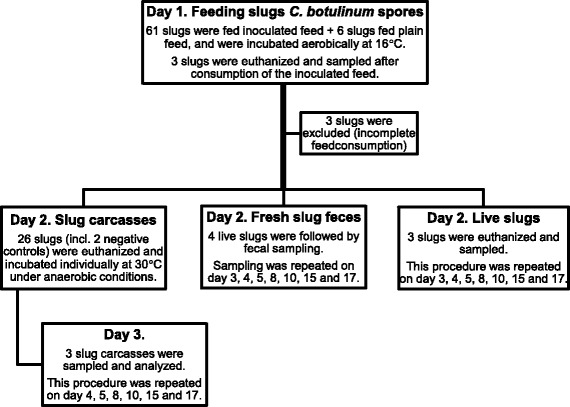
**Overview of the 17 day long feeding experiment.** Slugs were fed inoculated feed containing *C. botulinum* type C spores (or plain feed for negative controls) on day one, followed by sampling of slug carcasses, fresh slug feces and live slugs.

The experiment included a total of 67 slugs, where six served as negative controls and seven as reserve (Figure 1). Negative controls of slugs or slug feces were sampled on each day of analysis. The slugs were weighed individually both at the start of the experiment and when they were euthanized.

To collect individual samples, the live slugs were kept individually in boxes. The boxes were changed daily (except in weekends) to reduce the risk of recontamination from the slugs’ own feces and mucus. The feed intake was monitored after 2, 4 and 24 h and slugs with incomplete feed consumption after 24 h were excluded. From the second day, the slugs were fed white cabbage (*Brassica oleracea convar. capitata var. alba*).

A day rhythm of 8 h LED light was controlled by a timer. The temperature was maintained at 16 ± 2°C throughout the experiment for the live slugs, to simulate a mean Norwegian summer temperature. An incubation temperature of 30 ± 1°C was chosen for the slug carcasses. This temperature will support *C. botulinum* type C growth [5,22] and is relevant for newly made silage [23].

### Euthanizing of slugs and preparation of samples

Prior to analysis, slugs were rapidly euthanized by making a sagittal cut with a scalpel between the cephalic tentacles to cut the nerve ring in the head region of the slug. Slugs were finely chopped by scalpel prior to dilution in saline peptone water (dilution rate 1:10). A dilution rate of 1:100 was necessary for the slug feces samples, due to small feces amounts. The diluted samples were homogenized by hand for 2–4 min in BagFilter®’s (Interscience, St Nom la Bretêche, France). One ml of each sample was transferred into 1.5 ml tubes (Eppendorf AG). In addition, one ml was transferred to TPGY broth for enrichment. Inoculated TPGY broth was heated for 10 min at 70°C to eliminate growth of other than spore-forming bacteria before incubating at 30 ± 1°C for 72 h in anaerobic conditions. The heating of the TPGY broth would kill also vegetative *C. botulinum*, but those would be detected by qPCR in the non-enriched samples. To rationalize, not all TPGY-enriched samples were tested by PCR. Enriched samples from slugs and slug feces were only tested if the corresponding non-enriched sample yielded negative results and there was visible growth/gas-production in the TPGY broth. All samples were immediately frozen at −20°C and stored for up to two months before analysis by PCR.

### Detection and quantification of *C. botulinum* type C by PCR

A real-time PCR assay detecting the BoNTC gene covering a chimeric C/D sequence [24] was used [22]. The primers were F 5′-CACAAGAAGGATTTGGTGCTTTATCA-3′ and R 5′-CAGACTTAGAAAATCTACCCTCTCCTACA-3′ and the MGB (Minor Groove Binding) probe 5′-CATTACTATATGTTAGCATAAATCT-3′ with 5′ 6-FAM dye and a non- fluorescent quencher. The real-time PCR assay was made quantitative (qPCR) by generating a standard curve from the tenfold serial diluted and enumerated spore suspension of *C. botulinum*. The logarithm of the known initial concentrations plotted against the quantification cycle (Cq) value gives a straight line, and forms the standard curve [25]. The Cq values obtained from unknown samples were then converted to estimated cell numbers by this standard curve and the use of a software (Bio-Rad CFX manager 3.0).

DNA was extracted using Qiagen BioSprint 15 Blood kit (Qiagen, Hilden, Germany) and KingFisher mL for magnetic based separation (Thermo, Helsinki, Finland). The samples were centrifuged at 16 000 × g for 7 min. An amount of reagents as described for 200 μl of blood in the user manual was added to the sediments followed by the manufacturer’s instructions. The extracted DNA was analyzed with CFX96™ real-time PCR detection system (Bio-Rad Laboratories, Hercules, USA) or kept in a fridge at 4 ± 2°C for 18 h before analysis. Each run included a negative extraction control and positive and negative controls, in addition to the samples and the serial dilutions forming the standard curve. All samples, standards and controls were run in triplicates. To be considered as positive, two out of three Cq values had to be below 40. The qPCR mixture (25 μl) consisted of 12.5 μl LightCycler® 480 Probes Master (Roche Diagnostics GmbH, Mannheim, Germany), 0.45 μM of each primer, 0.3 μM of the fluorogenic probe (Applied Biosystems, Warrington, Great Britain), 0.25 μl Uracil-N-glycosylase (UNG, 1 U/μl, Eurogentec S.A., Seraing, Belgium), 6.25 μl RNase-free water and 3 μl of extracted DNA for testing. A multiplate 96-well format with adhesive sealing was used for the analysis (Bio-Rad).

### Statistics

Statistical analysis was performed with the Stata version 12 software packages (StataCorp LP, Texas, USA).

## Results

Six of 21 slug carcasses were positive for *C. botulinum* type C without the need of TPGY enrichment. The amounts were stable during the whole period (Table 1).

**Table 1:** **Quantitative detection of** ***C. botulinum*** **in slug carcasses by qPCR**

**Day**	**N positive**	**Mean Cq** ^**a**^ **(SD)**	**Mean log CFU/g (SD)**
3	0 (of 3)	ND	ND
4	1 (of 3)	39.15 (±0.72)	1.4 (±1.1)
5	1 (of 3)	38.39 (±1.01)	1.7 (±1.5)
8	1 (of 3)	38.97 (±0.63)	1.6 (±1.2)
10	1 (of 3)	39.79 (±0.05)	1.3 (±≅ 0)
15	0 (of 3)	ND	ND
17	2 (of 3)	38.9 (±0.07)	1.6 (±0.03)

^a^Mean Cq represents the mean of the positively quantified samples. Each sample was run in three qPCR replicates. The corresponding mean log CFU/g was calculated from the standard curve.

qPCR, Quantitative real-time PCR.

Cq, Quantification cycle.

SD, Standard deviation.

CFU, Colony-forming units.

ND, Not detected; less than 2 replicates per sample yielded Cq values <40.

*C. botulinum* type C was excreted in slug feces in declining numbers up to day four after the inoculated feed was given (Table 2). Cq values were markedly increased in enriched samples, proving viable *C. botulinum* (data not shown). Of the four slugs followed by fecal sampling, one slug was excluded due to producing too little or no feces for several days. Due to negative results, analysis of slug feces was terminated after day 8.

**Table 2:** **Quantitative detection of** ***C. botulinum*** **in slug feces by qPCR**

**Day**	**N positive**	**Mean Cq** ^**a**^ **(SD)**	**Mean log CFU/g (SD)**
2	3 (of 3)	35.3 (±2.13)	3.8 (±0.6)
3	1^b^ (of 3)	38.5 (±1.01)	2.9 (±0.7)
4	1^c^ (of 3)	39.0 (±0.60)	2.6 (±2.2)
5	0 (of 3)	ND	ND
8	0 (of 3)	ND	ND

^a^Mean Cq represents the mean of the positively quantified samples. Each sample was run in three qPCR replicates. The corresponding mean log CFU was calculated from the standard curve.

Additionally, 2^b^ (or 1^c^) other samples were positive after TPGY-enrichment (Cq not shown).

qPCR, quantitative real-time PCR.

Cq, quantification cycle.

SD, Standard deviation.

CFU, Colony-forming units.

ND, Not detected; less than 2 replicates per sample yielded Cq values <40.

**Figure 2:**
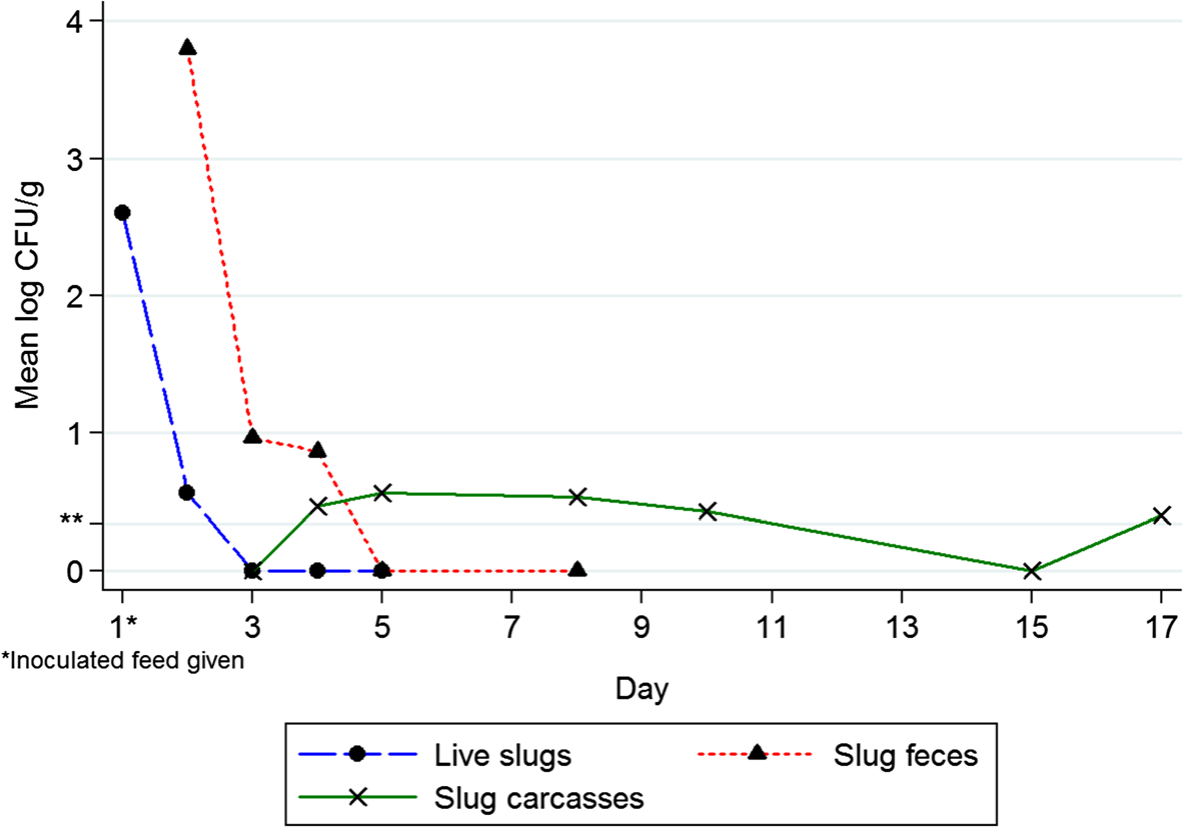
**Mean** ***C. botulinum*** **quantification in slug carcasses, slug feces and live slugs by qPCR.** Inoculated feed was given on day 1. The mean of three slug carcasses, three slug feces samples or three live slugs per day of analysis is included in the mean log CFU/g. Negative results are also included in the mean. **Detection limit >0.34 log CFU *C. botulinum*/g.

Live slugs were all positive for *C. botulinum* at day one, with a mean Cq of 36.0 (SD ± 1.3) and a corresponding mean log CFU/g slug of 2.6 (SD ± 0.4) for the three slugs. This gives a calculated CFU of 2.3 × 10^3^ *C. botulinum*/slug. At day two, one slug was positive and showed a mean Cq of 39.3 (SD ± 0.94) and 1.7 (SD ± 1.5) mean log CFU/g slug. The other two slugs were only positive after TPGY enrichment and with markedly increased Cq values. All live slugs tested negative during day 3–5. Figure 2 summarizes the mean quantification of *C. botulinum* in the slug carcasses, slug feces and live slugs. The pH measured from each of the five chopped slugs was pH 7.

### Standard curve and limit of detection

The standard curves generated acceptable efficiencies of 95.2%, 104.1%, 95.4%, slopes of −3.4, −3.2, −3.4 and R squares of 0.991, 0.995 and 0.990. They included 5 points of dilutions in three replicates. The most diluted of ≅ 2.2 CFU/ml showed replicates with Cq values above 40. Consequently a spore concentration above 2.2 CFU/ml (or 0.34 log CFU/g) was needed to be detected by this method. Samples with mean Cq values of ≥ 39 yielded SD above the recommended limit of 0.20 among replicates, which shows that the precision of quantification at such low levels is limited with this method. All negative control slugs in the feeding experiment and negative qPCR controls turned out negative for *C. botulinum* type C.

## Discussion

Both dead and live *A. vulgaris* showed to be poor growth substrates for *C. botulinum* containing the BoNTC gene in this feeding experiment. Nevertheless, viable *C. botulinum* were excreted in slug feces up to day four after the inoculated feed was given and persisted in the slug carcasses during the whole experiment.

*C. botulinum* has a broad ability to use different types of animal and vegetable proteins as a growth substrate [9,13]. In addition, colonization of the intestine is reported in infants, young animals and under special conditions also human and animal adults [5,9,10]. Surprisingly, our experiment showed that neither slug carcasses nor live slugs supported enrichment from the *C. botulinum* spores fed to the slugs. The bacterial number measured by this qPCR method is limited to the fraction of *C. botulinum* with intact BoNTC gene. This gene, located on a bacteriophage, can be lost during cultivation [26,27]. Still, visible growth or absence of growth in the TPGY broth supported the qPCR results, leaving no evidence of major gene-loss.

*C. botulinum* type C has a minimum growth temperature of 15°C. Although an optimum temperature of 40°C is reported, good growth conditions at 30°C are described [5,22]. A water activity over 0.98 and pH over 5.4 (terrestrial strains) or 5.1 (marine strains) are needed. These key parameters of growth were assumed fulfilled in our experiment, as measured from the five chopped slugs showing a pH of 7. James *et. al.* calculated mean pH in crop fluids from seven Black slugs (*A. ater*) to be 6.41± 0.33 (SE) [28]. Differences in the pH of the digestion system of starved and fed Gray (field) garden slug (*Deroceras reticulatum*) are reported, but all values were between pH 6 and 7 [29].

Germination of endospores is considered to be one of the most important limiting steps of *C. botulinum* enrichment [26]. The germination is still poorly understood and is triggered by complex interactions of nutrients, non-nutrients, enzymes and physical stimuli. Ensiling grass may add factors that might stimulate *C. botulinum* growth from contaminated slugs, given spots in the silage with interrupted pH decrease, but that was not investigated in the present study. Differences in the spore germination are seen between the four *C. botulinum* groups, but also individual strains within the same group show variability [26]. Thus, other *C. botulinum* strains may give different results than obtained in our experiment. On the other hand, *C. botulinum* is known to be a weak competitor in rich bacterial cultures like the intestinal microflora [30], and enrichment might have been inhibited by the bacterial flora of *A. vulgaris*. Slugs such as the closely related *A. ater,* and the keeled slugs *Limax maximus*, *Deroceras reticulatum* and snails such as *Cornu aspersum* and *Helix pomatia* are known to have a rich intestinal microflora. Bacteria belonging to *Enterobacteriaceae*, enterococci, lactobacilli and *Clostridium* spp. (none defined as *C. botulinum*) are among others reported from these slugs and snails [31–34]. In Sweden amounts of 5.4 log CFU/g of lactic acid bacteria and 6.0 log CFU/g *Enterobacteriaceae* are reported from *A. vulgaris* merged with slug feces and some grass residues [35]. Several intestinal bacterial strains, among them lactobacilli and enterococci, are reported to have bacteriostatic effects on *C. botulinum* [36–38].

Our experiment showed that slugs excreted viable *C. botulinum* type C in feces up to four days after orally being given a calculated amount of ≅ 5.8 (SD ± 0.1) × 10^4^ CFU *C. botulinum*/slug. The three slugs analyzed on day one showed that 2.3 ×10^3^ CFU/slug was successfully detected after feeding, a tenfold decrease compared to the given amount. The main reason of this difference was probably leakage of the spore suspension to the environment, since the slugs consumed the feed themselves. Low numbers of *C. botulinum* could be detected in slug carcasses during the entire period of testing. There was no evidence of enrichment. Hence the risk of classical botulism caused by BoNTC from slug contaminated silage would be considered low. Still, the actual effects of slugs into grass silage remain to be investigated. Well-preserved silage is protected by a pH below the growth limits of undesirable microorganisms like *C. botulinum*. However, an acidic pH will not destroy preformed neurotoxin [39]. The BoNT carrier status of *A. vulgaris* slugs feeding on contaminated carcasses would therefore be of interest. Fifteen of 37 aquatic snails collected on or close to avian carcasses tested positive for type C toxin with titers of 50–1600 mouse minimum lethal doses/g. None of 19 snails without the carcass association tested positive [15]. Slugs’ carrier status of BoNT is also relevant since slugs might be grazed directly. If slugs only hold a low spore contamination, without pre-formed BoNT, they would not be considered a greater risk to consume than feed from the already contaminated environment. The impact of large numbers of adult *A. vulgaris* slugs in preservation processes in silage remains to be investigated [35].

## Conclusions

Neither dead nor live invasive *A. vulgaris* slugs enriched *C. botulinum* containing the BoNTC gene in this study. Slugs excreted viable *C. botulinum* in feces up to four days after fed *C. botulinum* spores, but in rapidly decreasing numbers. *A. vulgaris* appear not to support *C. botulinum* type C enrichment.

## Abbreviations

BoNT: Botulinum neurotoxin
CFU: Colony-forming units
Cq: Quantification cycle
TPGY: Tryptose-peptone-glucose-yeast extract broth
qPCR: Quantitative real-time polymerase chain reaction
